# Molecular Mechanism of Autophagy and Its Regulation by Cannabinoids in Cancer

**DOI:** 10.3390/cancers13061211

**Published:** 2021-03-10

**Authors:** Xin Chien Lee, Evelyn Werner, Marco Falasca

**Affiliations:** Metabolic Signalling Group, Curtin Medical School, Curtin Health Innovation Research Institute, Curtin University, Perth, WA 6102, Australia; xinchienlee@curtin.edu.au (X.C.L.); evelyn.werner@student.curtin.edu.au (E.W.)

**Keywords:** cannabinoids, cannabinoid receptors, autophagy, cancer therapy, chemotherapy

## Abstract

**Simple Summary:**

This review examines the complex function of autophagy in malignancy and explores its regulation by cannabinoids in different cancers. Autophagy is an important process in the maintenance of cellular homeostasis, through the degradation and recycling of cytoplasmic constituents. The action of autophagy is highly dependent on tumour stage and type and the receptors with which ligands interact. Cannabinoids are growingly being acknowledged for their anticancer activities and are known to stimulate several mechanisms such as apoptosis and autophagy. Better understanding the mechanism of action behind autophagy and its regulation by cannabinoids will allow the development of novel cancer therapeutics.

**Abstract:**

Autophagy is a “self-degradation” process whereby malfunctioned cytoplasmic constituents and protein aggregates are engulfed by a vesicle called the autophagosome, and subsequently degraded by the lysosome. Autophagy plays a crucial role in sustaining protein homeostasis and can be an alternative source of energy under detrimental circumstances. Studies have demonstrated a paradoxical function for autophagy in cancer, displaying both tumour suppressive and tumour promotive roles. In early phases of tumour development autophagy promotes cancer cell death. In later phases, autophagy enables cancer cells to survive and withstand therapy. Cannabinoids, which are derivatives of the *Cannabis sativa* L. plant, have shown to be associated with autophagy induction in cells. There is an emerging interest in studying the signalling pathways involved in cannabinoid-induced autophagy and their potential application in anticancer therapies. In this review, the molecular mechanisms involved in the autophagy degradation process will be discussed. This review also highlights a role for autophagy in cancer progression, with cannabinoid-induced autophagy presenting a novel strategy for anticancer therapy.

## 1. Introduction

Autophagy is an intracellular degradation process that removes damaged organelles, misfolded proteins and non-functional protein aggregates [1]. In normal conditions, basal autophagy maintains cellular homeostasis, enhances cell growth and development, regulates immunity and inflammation, and acts as a defence mechanism against viral or bacterial infections [2,3,4]. Autophagy can be categorised into three pivotal groups; macroautophagy, microautophagy and chaperone-mediated autophagy, all promoting the proteolytic degradation of intracellular constituents by lysosomes [5]. Macroautophagy, hereby termed “autophagy”, entails the generation of double membraned vesicles, named autophagosomes, which assimilate unwanted organelles and proteins. This leads to the fusion of the autophagosome with lysosomes to form the autolysosome [5,6,7]. Lysosomal enzymes degrade the autophagic cargo, which is then recycled back to the cytosol to provide energy for cell growth [8]. In addition to the beneficial role for autophagy in maintaining normal cell growth, this process can also play a role in promoting cancer cell growth. Many studies have reported the dual action of autophagy, promoting either cell survival or cell death in cancer progression [9,10]. 

Cannabinoids are ligands that bind cannabinoid receptors and are classified into three groups: phytocannabinoids, endocannabinoids and synthetic cannabinoids [11]. Numerous recent studies have revealed that cannabinoids function as modulators in certain signalling pathways regulating cell proliferation and survival. Cannabinoids have been reported to induce apoptosis and autophagy pathways and inhibit tumour proliferation [12]. They have also shown potential for preventing tumour metastasis and angiogenesis [13]. Cannabidiol (CBD) and Δ^9^-tetrahydrocannabinol (THC) are the two most abundant phytocannabinoid compounds and are highly recognised for their therapeutic applications in anticancer therapies [14]. 

This review assesses the molecular mechanism and signalling pathways involved in the autophagy process, also exploring the anticancer action of cannabinoids, the cannabinoid-induced autophagy mechanism and the interplay between apoptosis and autophagy. It examines the dual role of autophagy in cancer, enhancing either cancer cell progression or death. In addition, this review highlights the potential use of cannabinoids and autophagy as targeted anticancer therapies. 

## 2. Molecular Mechanism of Autophagy

Autophagy depends on a sequence of dynamic membrane events. The process begins with sequestration of cytoplasmic components by a unique membrane, named the isolation membrane or “phagophore”. The phagophore undergoes elongation, resulting in a cup-shaped structure [4,15]. Sequestration is complete when the elongated phagophore is completely sealed and a double-membrane bound vesicle is formed, identified as the autophagosome. The autophagosome then fuses with the lysosome forming the “autophagolysosome”. The inner membrane of the autophagosome, the cytoplasmic constituents, and protein aggregates are then degraded by lysosomal hydrolases. The autophagolysosome turns into a leftover organelle, and breakdown molecules, including amino acids and nucleosides, are carried back to the cytoplasm and recycled as chemical energy or building blocks for other cellular mechanisms [1,16]. 

**Figure 1:**
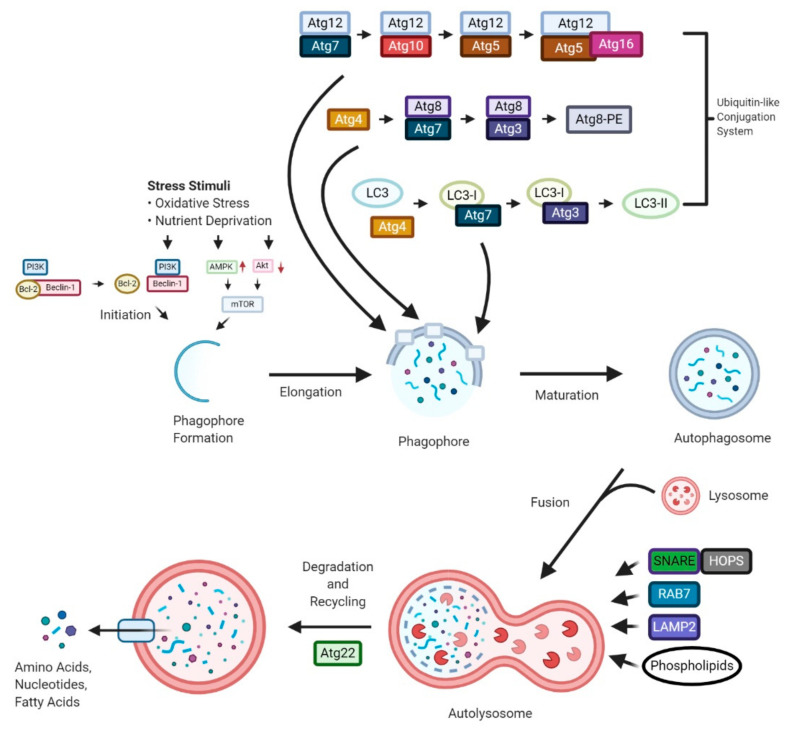
Molecular Mechanism of Autophagy. Schematic model of autophagy steps showing the different environmental conditions and molecular machinery regulating its progression. LC3, microtubule-associated protein light chain 3; AMPK; adenosine monophosphate-activated protein kinase; PI3K, phosphoinositide 3-kinase; Akt, protein kinase B; ATG, autophagy-related; mTOR, mammalian target of rapamycin; Bcl-2, B-cell lymphoma 2; SNARE, soluble *N*-ethylmaleimide-sensitive factor attachment protein receptor; RAB7, Ras-related protein 7; LAMP2, Lysosome-associated membrane protein 2; HOPS, homotypic fusion and vacuole protein sorting.

Distinct sets of autophagy-related (Atg) proteins are involved in the various stages of autophagy, either individually or in combination (Figure 1). Many autophagy studies have reported a highly conserved mechanism of autophagy from yeasts to higher eukaryotes. There are a total of 31 Atg proteins that have been discovered in yeast [17]. 

Mizhushima et al. found presence of Atg12, Atg5 and Atg16 homologs in humans, acting in a similar mechanism to what is seen in yeast [18]. The study showed the binding of hAtg12 with hAtg5, then two conjugated hAtg12-hAtg5 complexes forming a dimer with hAtg16L, aiding in the phagophore elongation in human autophagy [18]. Microtubule-associated protein light chain 3 (LC3B) is another ubiquitin conjugation system that was identified and is a mammalian homolog of Atg8. LC3B-I, a processed form of LC3B, is catalysed by the cysteine protease hAtg4, the mammalian homolog of Atg4 [19]. E1 ubiquitin-activating enzyme Atg7, interacts with the C-terminal glycine residue of LC3B-I. The activated LC3B-I is then transferred to the E2 enzyme Atg3, thereby driving the conversion of LC3B-I to LC3B-II [19,20,21]. Like the Atg8-PE conjugation system found in yeast, the hAtg12-hAtg5 conjugates initiate the incorporation of LC3B-II to the elongating phagophore. LC3B-II was reported to be localized at both the inner and outer autophagosome membranes and, hence, LC3B-II is known as a specific and reliable marker for the autophagosome [19]. LC3B is also a receptor for the selective substrate p62/SQSTM1, which serves as a target for organelles and protein aggregates, resulting in selectively induced autophagy. p62/SQSTM1 is degraded by lysosomes with the cytoplasmic cargo and its decreasing levels indicate the presence of autophagy, making it another autophagy marker. In autophagy-deprived cells, the accumulation of p62/SQSTM1 is observed [22]. 

Along with the ATG genes and their homologs, various autophagy studies have also focused on molecular machinery involved in the fusion stage of autophagy. SNARE (soluble *N*-ethylmaleimide-sensitive factor attachment protein receptor) proteins, tethering factors and lipids are involved in autophagosome-lysosome fusion [23]. Several factors modulate the assemblage of the autophagosomal SNARE complex. For this to happen, both the autophagosome and lysosomes need to be physically close enough to tether prior to the SNARE-mediated fusion. The first step of SNARE-mediated fusion is the assembly of R-SNARE and Q-SNARE proteins into the trans-SNARE complexes, to ensure a source of energy for fusion [24]. In mammalian structures STX17, the autophagosomal Q-SNARE, is employed from the mitochondria and endoplasmic reticulum (ER) to completed autophagosomes when autophagy is induced [25].

In addition to SNAREs and tethering factors, phosphoinositides also play a pivotal role in the modulation of the cell signalling and managing the membrane modelling. To date, there are three phosphoinositides, phosphatidylinositol 3-monophosphate, phosphatidylinositol 4-monophosphate and phosphatidylinositol 3,5-bisphosphate, that have been reported to exert a role in autophagosome-lysosome fusion [26,27].

## 3. Regulation of Autophagy

Autophagy plays a fundamental role in various cellular mechanisms and its dysfunction can lead to the progression of a number of human diseases. Nutrient deficiency has been reported as one of the factors that induces autophagy. Autophagy is initiated by intermittent fasting and is repressed by food consumption, highlighting a tight regulation of autophagy by the nutritional state of cells. According to early studies, approximately 30–40% of liver proteins are degraded after 48 hours of starvation, and Mortimore et al. revealed that amino acids released from the degraded proteins play a role in the regulation of autophagy [28,29,30]. Deprivation of amino acids in a perfused rat liver improved the rate of protein degradation dramatically while increased levels of amino acids decreased the rate of protein degradation [31]. Despite total levels of amino acids affecting the induction or inhibition of autophagy, individual amino acid levels can also affect the progression of autophagy. Amino acids leucine, tyrosine, phenylalanine, alanine, glutamine, proline, histidine, tryptophan and methionine displayed inhibitory effects on autophagy when acting individually in the perfused rat liver [30]. The nutrient regulation of autophagy has also been reported to be mediated by hormones and growth factors. Autophagy in the liver is induced by glucagon and is supressed by insulin [32]. Autophagy is also suppressed by the hematopoietic growth factor interleukin-3 (IL-3) via the regulation of nutrient accessibility [33]. Amino acids, hormones and growth factor signals are believed to congregate on the mammalian target of rapamycin (mTOR), which is a key regulator of nutrient signalling pathways. Target of rapamycin (TOR) inhibitors such as CCI-779 and rapamycin were found to induce autophagy in yeast and animals [34,35]. Reagents such as chloroquine (CQ) and bafilomycin, however, elevate lysosomal pH and obstruct autophagic protein degradation. Despite the fact that these compounds alter the acidity of lysosome pH, they largely influence a variety of cellular functions, thus limiting the practicality of these findings [1].

Another regulator of autophagy is Beclin-1, which is one of the most well studied mammalian-specific autophagy regulators and is the mammalian homologue of yeast ATG6. It provides a platform for the recruitment and initiation of the class III phosphoinositide 3-kinase (P13K) complex [36]. In normal mammalian cell growth conditions Beclin-1 binds with Bcl-2, an anti-apoptotic protein. This occurs via interaction through a Bcl-2 homology 3 domain (BH3) in Beclin-1, preventing the formation of the Beclin-1/class III PI3K complex, resulting in autophagy inhibition [37]. In contrast, Bcl-2 is released from Beclin-1 when exposed to nutrition deficient conditions, and autophagy is induced [38]. 

## 4. Autophagy Signalling Pathways

**Figure 2:**
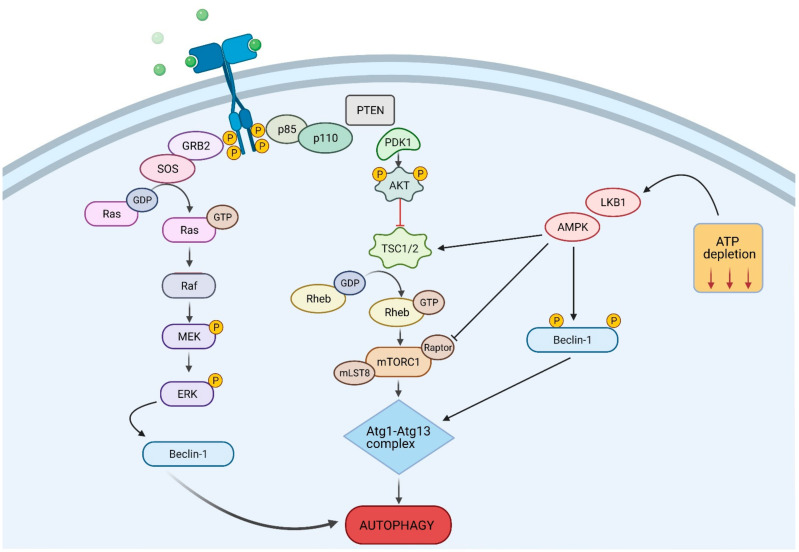
Autophagy Signalling Pathways. Multiple signalling pathways, some of which are shown in the figure, regulate autophagy. The main players are the mTOR kinase, that activated by growth signals suppresses autophagy, and Beclin-1, that activated by stress signals induces the formation of the phagophore membrane. GRB2, Growth factor receptor-bound protein 2; SOS, Son of sevenless; p85, Phosphoinositide-3-Kinase Regulatory Subunit; p110, Phosphoinositide-3-Kinase catalytic Subunit; PDK1, Phosphoinoisitide-dependent kinase-1; PTEN, Phosphoinoisitide dependent kinase; TSC1/2, Tuberous sclerosis proteins 1 and 2; RHEB, Ras homolog enriched in brain; Raptor, Regulatory-associated protein of mTOR; mLST8, mammalian lethal with SEC13 protein 8; LKB1, liver kinase B1; Raf, Rapidly Accelerated Fibrosarcoma; MEK, Mitogen-activated protein kinase kinase; ERK, extracellular signal-regulated kinase.

There are multiple signalling pathways through which autophagy can be activated (Figure 2). mTOR is a negative regulator of autophagy and plays an essential role in cellular processes such as protein synthesis, cell cycle and cell proliferation. It is modulated by upstream effectors PI3K and protein kinase B (AKT). AKT is a serine/threonine kinase that is important for cell metabolism, growth, proliferation and survival. Activation of the PI3K/AKT/mTOR signalling cascade by extracellular stimuli leads to suppression of the autophagy pathway and a pro-tumorigenic action. PI3K phosphorylates AKT, which in turn phosphorylates mTOR to block autophagy induction. Alternatively, inhibition of the PI3K/AKT/mTOR axis leads to the activation of autophagy [39]. 

Adenosine monophosphate-activated protein kinase (AMPK) is another pathway through which autophagy is regulated. AMPK promotes autophagy induction through the inhibition of mTOR. Under metabolic stress, AMPK activates ULK1, the human homologue of yeast ATG1, and phosphorylates tuberous sclerosis protein 2 (TSC2) to inhibit mTOR and activate autophagy [40,41].

Mitogen-activated protein kinases (MAPKs) pathways give rise to another diverse and important regulatory mechanism involved in a wide variety of cellular processes including cell proliferation, differentiation, transformation, inflammation and apoptosis. MAPKs are separated into three main subfamilies: p38, Jun *N*-terminal kinase (JNK) and extracellular-regulated kinase (ERK) and are activated in response to extracellular stimuli such as stress, hormones and growth factors. Activation of p38, JNK and ERK MAPK pathways inhibits the induction of autophagy, through phosphorylation of ULK1 [42,43]. 

Each of these signalling pathways plays important roles in cell proliferation and has been implicated in tumorigenesis; thus, they are increasingly looked at as potential therapeutic targets, suggesting a role for autophagy in cancer therapy.

## 5. The Role of Autophagy in Cancer

In cancer cells, autophagy can either exhibit a tumour suppressive role or can aid in tumour growth and survival. There are many factors that influence its function, including tumour developmental states, tissue and cellular microenvironments, and the duration of stress-activating stimuli, which contribute to regulation of autophagy in cancer cells [9,44,45]. In early stages of tumorigenesis, autophagy demonstrates antitumorigenic properties reducing tumour invasiveness and inhibiting cancer cell growth by limiting necrosis and the inflammatory response. A pro-metastatic role of autophagy is noticeable; however, at advanced stages of malignancy, autophagy acts as an alternative source of energy under stressful tumour microenvironments, thus enhancing cancer cell survival [44,46,47]. Due to the complicated role of autophagy in cancer, there is an emerging interest in investigating its potential as a therapeutic target in cancer treatment as well as its possible application in combination therapy with existing therapeutic agents. 

### 5.1. Autophagy and its Tumour Suppressive Role

An antitumor action of autophagy has been found through the investigation of the tumour suppressor gene BECN1, which encodes the known autophagy promoter Beclin-1 [48]. Loss of the BECN1 gene is observed in numerous cancers including human ovarian, breast, and prostate cancers [36,49,50]. Mutated BECN1 results in the reduction of autophagy and augmentation of cell proliferation in cancer cell lines and mice models, suggesting that BECN1 aids in tumour regression [51,52]. Several studies have reported that BECN1^±^ mutant mice models are more prone to develop tumours as compared to BECN1^+/+^ wild type mice, particularly lymphoma, liver and cervical tumours [51,52,53]. Depletion of Beclin-1 is also traceable in various human brain tumours [54]. The interaction between Beclin-1 and other proteins, such as Bax interacting factor-1 (Bif-1), UV radiation resistance-associated gene (UVRAG) and BECN1-regulated autophagy protein 1 (Ambra1), have been found to stimulate the class III PI3K, which positively regulates autophagy [55,56,57,58]. 

p62, also termed sequestosome-1 (SQSTM1), is a multifunctional protein chaperone that is involved in numerous signalling mechanisms including autophagy. p62/SQSTM1 acts as a crucial cargo receptor to recognise and transport aberrant protein aggregates and damaged organelles to the autophagosome for degradation [59]. Recent research has focused on the role of p62/SQSTM1 in tumorigenesis. Accumulation of p62 results in elevated ER stress and DNA destruction [60]. Expression of p62/SQSTMQ1 is shown to promote tumorigenesis and augmented levels of p62/SQSTM1 are frequently found in various human cancers including pancreas, prostate, liver and lung cancers [61,62,63]. With regard to autophagy, p62/SQSTM1 functions as an adaptor protein that induces the interaction between LC3 and ubiquitin moieties on misfolded proteins. The clearance of p62, together with the ubiquitinylated proteins, is therefore facilitated by autophagy. Hence, autophagy inhibition leads to accumulation of p62/SQSTM1 and contributes to tumorigenesis [60]. An in vivo study by Takamura et al. has shown that mouse models with deletion of Atg proteins, such as Atg5 or Atg7, have exhibited an accumulation of p62 in the grown tumours [64]. Furthermore, overexpression of p62/SQSTM1 in hepatocytes is able to mediate oncogenesis, as p62/SQSTM1 is a major component of Mallory bodies, which accumulate in the human hepatocellular carcinoma [62,65]. p62/SQSTM1 deficiency in cancer cells significantly impedes cancer cell growth and inhibition of p62/SQSTM1 suppresses KRAS-driven lung cancers in a genetically engineered mouse model [61]. Paradoxically, it has been seen that the upregulation of p62/SQSTM1 is associated with human tumour progression and downregulation induces cancer progression in cancer-associated fibroblasts [66,67]. An in vivo study by Huang et al. has proposed that the selective inactivation of p62/SQSTM1 in adipocytes increases osteopontin levels, boosting fatty-acid oxidation in cancer cells and eventually leading to the development of invasive metastatic prostate cancers [68]. Therefore, the quality control role played by autophagy, through the removal of damaged proteins and organelles, maintains genome stability and prevents tumour initiation, avoiding cell injury and chronic tissue damage, and impeding the formation of oncogenic p62 protein aggregates.

### 5.2. Autophagy and Its Tumour Promoter Role

Induction of autophagy is an adaptive mechanism for cells that are metabolically stressed. Although autophagy drives antitumorigenic action in the initial stages of cancer development, tumour cells are frequently and continuously exposed to detrimental stress stimuli, such as hypoxia, inflammation and nutrient deprivation [69]. Hypoxic conditions have been reported as a hallmark of many cancer microenvironments [70]. When cancer cells are entering the dormancy period, the activation of autophagy serves as a survival strategy. Hence, hypoxia-mediated autophagy is induced to fulfil the energy demand for tumour cell maintenance, thereby increasing the cell survival rates under extreme stress circumstances [71]. The hypoxic cancer microenvironment induces autophagy through initiation of the stress response signalling mechanism hypoxia-induce factor-1 alpha (HIF-1α), which alleviates the energy deprivation, thus enhancing the cancer progression and survival [72]. Investigation by Denko et al. has shown enhanced glucose metabolism via the HIF-1α signalling pathway and consequent promotion of autophagy [73]. Stimulation of AMPK and the inhibition of mTOR is another pathway employed to activate autophagy when cancer cells undergo amino acid and glucose deficiency [74]. Along with this, a crosstalk between inflammation and autophagy in cancer cell microenvironments has been mentioned in various studies [75]. Inflammation is shown to reduce autophagy stimulation in cancers, whereas autophagy defects in tumours are found to trigger inflammation that may generate pneumonia [76,77,78]. 

Autophagy is initiated in Ras-activated cancer cells and consequently encourages cancer cell growth, development, invasiveness and metastasis. Ras are small GTPases that aid in regulating tumour proliferation and survival through several signalling pathways [79]. KRAS and HRAS are two well-known oncoproteins that upregulate autophagy as a pro-survival mechanism for cancer cells to overcome the metabolic stress [80]. KRAS^G12D^-driven lung cancer, as an example, uses autophagy as an essential mechanism for maintaining mitochondrial function and tumour cell survival under the stressful environment. Hence, the inhibition of autophagy impedes tumour cell growth and modulates the conversion of adenomas and carcinomas to benign oncocytoma-like tumours [81]. In genetically engineered mouse models (GEMMs) the deletion of essential Atg7 is associated with KRAS-driven non-small-cell lung cancer (NSCLC). Atg7 deficiency in tumours leads to malfunction of the mitochondria and activation of tumour-suppressor p53. This finding has indicated that loss of Atg7 impairs the autophagy pathway, leading to augmentation of p53 and suppression of tumour growth, extending the life span of a mouse model [81]. It has been suggested that p53 loss particularly increases glycolysis and diminishes the oxidative metabolism in a KRAS-driven pancreatic model, alleviating the necessity of autophagy for energy production [82]. The reliance on autophagy and p53 in tumour survival is largely dependent on the cell types in cancer [82,83]. Despite the exhibition of a pro-survival role by Ras-mediated autophagy, HRAS-activated autophagy induces caspase-independent cancer cell death [84]. Along with the Ras-driven cancers, autophagy plays an oncogenic role in BRAF-driven melanoma. Specific Atg7 and Atg5 ablation of BRAFV600E-driven melanoma inhibits tumour formation and induces melanocyte senescence in GEMMs [85]. Another study by Liu et al. discovered that Atg5, the pivotal autophagy-regulating gene, is significantly downregulated in primary melanomas, proving that reduced autophagy promotes tumour progression and decreases survival rate in early phase cutaneous melanoma patients [86].

In addition to its pro-tumorigenic role, autophagy has also been demonstrated to exert a pro-metastatic one. In order to become invasive and facilitate the colonisation of secondary sites, cancer cells must be able to overcome anoikis, which is defined as the apoptotic cell death that occurs when cells lose attachment from extracellular matrix (ECM) [87,88]. Cancer cells elude anoikis and survive via abnormal stimulation of certain autophagic signalling pathways, including the Ras-driven mechanism, PI3K/AKT pathway, and the ERK signalling route [44,89]. Emerging discoveries have demonstrated that protective autophagy contributes to anoikis resistance in tumour cells. Recent studies have reported that the inhibition of the β1-integrin receptor induces autophagy and enhances ECM detachment [89]. Another study indicated that the inhibition of autophagy reduced anoikis resistance and hindered the metastasis of hepatocellular carcinoma (HCC) associated in a lung metastasis model [90]. A number of studies reported that the augmented level of autophagy was found in epithelial mesenchymal transition (EMT)-induced cancer cells, allowing them to withstand the stressful microenvironment of the metastatic phase in cancer [91]. It has been illustrated that the decreased expression of key autophagy regulator proteins, including ATG5, ATG7, and Beclin-1, enhance the invasiveness and migration with EMT regulators in glioblastoma cells [92]. Moreover, the EMT induced by a type 2 cadherin, known as cadherin-6, in embryonic development is aberrantly heightened in cancer and enhances metastasis [93]. 

In brief, during tumour progression autophagy favours survival and proliferation of the growing tumours by eliminating damaged proteins and toxic radicals, preventing mitochondrial malfunction, and facilitating cancer metabolism. Furthermore, autophagy contributes to the metastatic process and may affect the efficacy of many chemotherapeutic agents.

### 5.3. Autophagy as a Therapeutic Target in Cancer

The role of autophagy in anticancer therapies has been extensively studied in recent years. While acting as a protective mechanism for tumour cells, upregulation of autophagy enables tumour cells to develop resistance to a wide range of anticancer therapies [94]. Conventional chemotherapeutic agents, such as gemcitabine and temozolomide, have been the most effective methods of cancer treatment before cancer cells exhibited resistance to these anticancer drugs. Despite its effectiveness in eliminating cancer cells, the high cytotoxicity of conventional chemotherapy has been accompanied by numerous adverse effects such as nausea and emesis and the chemoresistance that develops in tumour cells has restricted the success rate of chemotherapy [94]. 5–Fluorouracil (5FU) is one of the anticancer drugs that is commonly used in treating numerous human cancers, including pancreatic, colorectal and breast cancer [95]. 5FU represses thymidylate synthetase, which is followed by the inhibition of DNA synthesis [96]. Chemoresistance activated by protective autophagy in various tumour cells has limited the effectiveness of treatment with 5FU. Beclin-1 expression is mediated by protective autophagy, subsequently leading to the conversion of LC3-I to LC3-II, which drives the maturation of the autophagosome. JNK-facilitated protective autophagy and augmented levels of B-cell lymphoma 2 (Bcl2) surges autophagic flux, thus triggering chemoresistance [96,97,98]. Another anticancer drug, cisplatin, displays its treatment efficacy by stimulating the impairment in DNA and interfering with the mitochondrial apoptosis signalling pathways. Similar to 5FU, chemoresistance development in tumour cells lowered the success rate of cisplatin treatment in human cancers [99]. One study has reported that protective autophagy is induced through the overexpression of Beclin-1 and the regulation of ERK, resulting in treatment resistance in ovarian cancer [100,101]. Additionally, cisplastin treatment efficacy is limited in oesophageal cancer, as protective autophagy is enhanced through increased expression of Beclin-1, augmented level of ATG7, and the transition from LC3-I to LC3-II [102,103]. Therefore, more investigations should be conducted to look for approaches to overcome chemoresistance through the regulation of autophagy signalling pathways and autophagy levels present in cancer cells, thus restoring the efficacy of anticancer therapies.

The regulation of autophagy serves as a promising potential treatment strategy in enhancing cancer therapy. Studies have reported that accumulation of autophagosomes indicates upregulation of autophagy in response to treatments with conventional anticancer drugs such as temozolomide, newly targeted cancer therapies such as tamoxifen or the exposure to ionising radiation. These findings suggest that autophagy exhibits both pro-survival and pro-death roles in response to anticancer therapies [104]. DNA damaging agent camptothecin (CPT) has been shown to induce protective autophagy and inhibit cell death in breast cancer cells [105]. Activation of protective autophagy by 5FU results in drug resistance by oesophageal and colon cancers [106]. Inhibition of protective autophagy therefore plays a crucial role in increasing the efficacy of the anticancer therapies. Autophagy inhibitors such as CQ and hydroxychloroquine (HCQ) are widely used as both monotherapies and combination therapies with existing conventional anticancer drugs [106,107]. Both CQ and HCQ alter lysosomal pH, thus disrupting the turnover phase of autophagy [108]. Evidence has revealed the efficacy of CQ and HCQ in hindering cancer cell proliferation through autophagy inhibition in pancreatic ductal adenocarcinoma and bladder cancers [109]. HQL is not effective as a monotherapy but shows improvement when used in combination with gemcitabine with nab-paclitaxel. Parallel inhibition of ERK synergistically enhances HQL inhibition. Inhibiting ERK impairs metabolic processes such as glycolysis and mitochondrial function, which leads to a dependence on autophagy for the tumour to progress. Pairing that with HQL inhibition of autophagy leads to an enhanced efficacy of treatment [110]. HCQ is also able to sensitize tumour cells to 5FU treatment in colorectal cancer [111]. Another autophagy inhibitor, 3-methyladenine (3-MA), exhibits a similar effect in combination treatment with 5FU in colorectal cancers [104]. 

More recent studies have reported several novel autophagy inhibitors for cancers. Lys05, for instance, is a water-soluble analogue of HCQ that is able to inhibit autophagy via the impairment of lysosomal function, even at a low dose [112]. In vitro and in vivo studies have demonstrated that Lys05 has a higher anticancer efficacy as compared to HCQ in colon cancer and melanoma xenograft models [113]. Combination therapy involving Lys05 and BRAF inhibitors has shown to inhibit cancer cell growth in vivo [114]. Furthermore, SAR405, a highly potent class III PI3K inhibitor, inhibits autophagy by altering lysosomal function [115]. Studies have reported that combination therapy of SAR405 with the mTOR inhibitor everolimus promotes the inhibition of cancer progression in renal tumour cell lines, hence indicating the efficacy of SAR405 as an anticancer drug that targets the regulation of autophagy [116]. In addition to autophagy and proteasome inhibitors, other autophagy-related drugs have been introduced for cancer treatment. Spautin-1 suppresses autophagy and results in the stimulation of proteasomal elimination of class III PI3K multiplexes [117]. It exhibits a pro-apoptotic effect that is associated with GSK3β and influences the downstream target of the PI3K/AKT signalling mechanism, thus is also recognised as a potential therapeutic agent in anticancer therapies [117]. 

## 6. The Endocannabinoid System

The endocannabinoid system plays a crucial role in modulating several physiological processes and is generating interest for the linkage of its dysregulation to several pathologies [118]. Cannabinoids are bioactive lipids that interact with cell-surface receptors [119]. Endocannabinoids are endogenous ligands produced by the body to bind cannabinoid receptors [119]. They include anandamide or *N*-arachidonoylethanolamine (AEA) and 2-arachidonoylglycerol (2-AG) [120,121]. There are also endocannabinoid-like substances, *N*-palmitoylethanolamine (PEA) and *N*-oleoylethanolamine (OEA), which are fatty acid amides [122]. Phytocannabinoids are derived from the *Cannabis sativa* L. plant and include THC, CBD and around 100 others [123,124]. Cannabis is the most widely consumed illicit drug, with its active components found in the stalks, leaves, flowers and seeds of the plant [124]. Synthetic cannabinoids are developed in a laboratory and include WIN-55,212-2, JWH-105 and arachidonyl-2′-chloroethylamide (ACEA) [125]. They have similar properties and action to endocannabinoids and phytocannabinoids, however, they can be synthesized to be more potent and selective.

The two known ‘canonical’ cannabinoid receptors, cannabinoid receptor 1 (CB1) and cannabinoid receptor 2 (CB2), are part of the G-protein coupled receptor (GPCR) family [126,127]. Their activation inhibits adenylyl cyclase via the Gi protein and leads to the activation of a number of downstream physiological and pathological pathways [128]. CB1 is predominantly expressed in the central nervous system (CNS) and its activation has psychoactive effects [129]. CB2 is expressed in the immune system and has a protective role [130]. THC, the psychoactive component of cannabis, engages both CB1 and CB2 (107). Endocannabinoids AEA and 2-AG are also agonists, with AEA having a higher affinity for CB1 and 2-AG having a higher affinity for CB2 [131]. CBD has low affinity for CB1 and CB2 and instead interacts with other receptors that are considered non-canonical cannabinoid receptors. These include G-protein coupled receptor 55 (GPR55), transient receptor potential vanilloid type 1 (TRPV1) and type 2 (TRPV2), and peroxisome proliferator-activated receptors (PPARs) [126,127]. CBD acts as an antagonist of GPR55, binding to the receptor to block its signalling [132]. Endocannabinoid-like substances PEA and OEA have low affinity for CB1 and CB2 but are PPAR agonists [133]. Along with the ligands and receptors, the endocannabinoid system also consists of metabolising enzymes such as fatty acid amide hydrolase (FAAH) and monoacylglycerol lipase (MAGL), which are involved in hydrolysis and degradation of endocannabinoids AEA and 2-AG, respectively [134,135].

## 7. Anticancer Properties of Cannabinoids

Phytocannabinoids boast a centuries-long history of medicinal use; however, they have been recently put in the spotlight following scientific studies indicating their potential therapeutic efficacy in a variety of areas, including cancer. For a long time, cannabinoids were used as palliation for chemotherapy side effects and cancer symptoms; however, more information is emerging surrounding the anticancer properties of cannabinoids [13,136]. There is a large amount of data suggesting cannabinoids exert an inhibitory effect on cancer cell proliferation [12]. Cannabinoid receptors and their ligands are upregulated in cancer cells [137,138]. As discussed above, overexpression of cannabinoid receptors CB1, CB2 and non-canonical receptors such as GPR55, as well as endocannabinoid metabolising enzymes FAAH and MAGL, correlates with tumour aggressiveness and indicates the importance of the ECS in cancer progression [139,140]. Nevertheless, the clinical studies are limited and contrasting results have emerged for different types of cancer. The direct and indirect anticancer activity of cannabinoids, independent from the interaction with cannabinoid receptors, has also been studied. These antitumour functions may involve alterations of cell signalling pathways resulting in decreased cancer cell proliferation, apoptosis and inhibition of migration, or affecting tumour vascularization, microenvironment, immune response, and inflammation. Despite the large amount of work on anticancer activities of cannabinoids, the majority of studies have been performed in vitro and in xenograft animal models. Consequently, there is a limited number of investigations in more complex models such as transgenic animals where the tumour architecture and the involvement of the tumour microenvironment and immune response can be recapitulated making them representative of patients’ pathological situation. The majority of preclinical evidence available so far demonstrates that cannabinoids’ greatest therapeutic potential resides in their combination with existing chemotherapy drugs. However, despite all the studies, many questions remain open. Aside from the issues of formulation, absorption and bioavailability, other unresolved problems undermine cannabinoids’ progression towards their therapeutic use in cancer. 

Increasing evidence points to the tumour microenvironment as an essential player in cancer growth and progression. This particular “habitat” is composed of a variety of constituents, such as fibroblasts, endothelial and immune cells, and extracellular matrix, which are manipulated by tumour cells for the purpose of facilitating their progression [141]. As the majority of cells composing the tumour microenvironment possess cannabinoid receptors, it has not been clarified yet whether cannabinoids’ activity may be promoting tumour development or having an antitumour effect. Inflammation, caused by either external elements or by intrinsic tumour-related factors, is another well-known hallmark of cancer promoting immunosuppression and contributing to tumours’ rise and evolution [142]. Even though some studies have highlighted cannabinoids’ potential as anti-inflammatory drugs, the role of cannabinoids in the regulation of inflammation has not been fully elucidated. Recently immunotherapy, the use of patients’ own immune system for identifying and suppressing cancer cells, has seen increasing attention [141]. Data collected so far on cannabinoids, especially on THC, seem to point towards a negative effect on lymphocytes, leaving unanswered the question about their role in aiding or inhibiting the immune response [143]. 

Currently, the main approved application of cannabinoid-based medications remains palliative care and the treatment of side effects such as nausea and anorexia or as anti-spastics in some neurological conditions. In regards to the potential use of cannabinoids and derivatives in cancer therapy, many unanswered questions for researchers and clinicians need to be addressed. Among these, the effect of cannabinoids on tumour microenvironment and immune response are pivotal [144]. In particular, the potential negative effect exerted by cannabinoids on immune response raises concerns regarding their clinical use [145]. 

**Figure 3:**
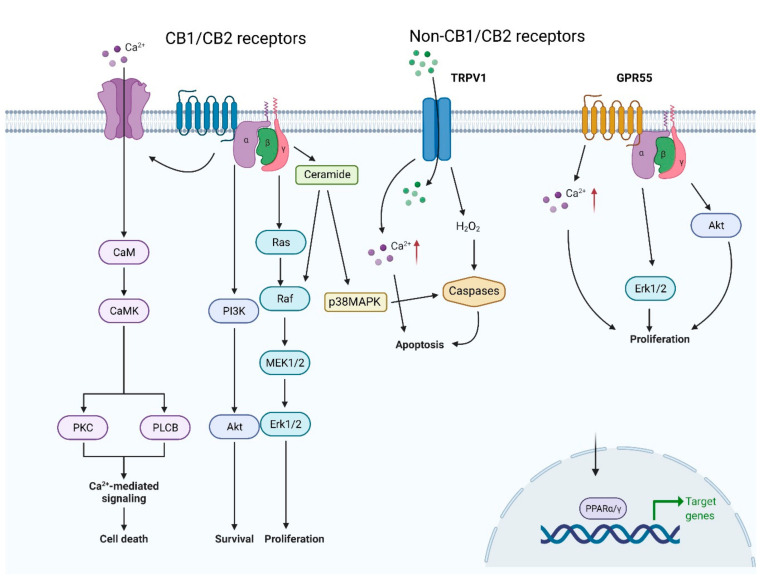
Endocannabinoid Signalling. The figure shows the main receptors activated by endocannabinoids and the corresponding signalling pathways initiated by them. CB1/2, cannabinoid receptor 1/2; CaM, calmodulin; CaMK, calmodulin-dependent protein kinase; PKC, protein kinase C; PLCB, phospholipase C beta; p38MAPK, p38 mitogen-activated protein kinase; TRPV1, transient receptor potential vanilloid type 1; GPR55, G-protein coupled receptor 55; PPARα/γ, peroxisome proliferator-activated receptors α/γ.

Binding of ligands to cannabinoid receptors activates a number of downstream pathways that can inhibit cancer cell proliferation and lead to cancer-cell death by apoptosis (Figure 3). This has been displayed in a variety of cancer types including glioma, melanoma, pancreatic and breast cancers [146,147,148,149]. 

The P13/AKT/mTOR axis, a commonly dysregulated pathway in cancer, is responsible for the regulation of cell growth and survival, and is one pathway activated by the binding of cannabinoids to their receptors [150]. The binding of THC to receptors in glioma causes accumulation of ceramide which puts stress on the ER and leads to pseudokinase tribbles homologue-3 (TRIB3)-dependent inhibition of AKT/mTOR [146]. This then inhibits the proliferation of glioma cells. Similarly, in carcinoma cells, cannabinoids JWH-015 and THC inhibit AKT/mTORC1 through ER stress-dependent activation of AMPK [149]. When this is blocked, cancer cells continue to grow [149]. Another pathway activated by cannabinoids, which is involved in reducing cancer cell proliferation, is the MAPK pathway. These serine/threonine protein kinases convert stress stimuli into different cellular responses such as cytokine production, cell cycle arrest and cell death, via phosphorylation of specific targets [151]. The treatment of cells with GPR55 antagonists CID16020046 and CBD prevents MAPK signalling, cell growth and cell cycle progression [133]. This is also seen when CB1 and CB2 receptors are activated by THC or synthetic cannabinoids WIN-55,212-2, CP55940, JWH-133 or HU210, and accumulation of ceramide and ER stress alters MAPK signalling, inhibiting glioma cell growth [152]. The activation of cannabinoid receptors by their ligands leads to a number of downstream pathways that inhibit cancer cell proliferation, as well as having other anticancer effects such as cell cycle arrest and cell death. It is important to understand better the pharmacological activities of cannabinoids and the mechanisms behind them, to know how to utilise them therapeutically.

### 7.1. Cannabinoids Modulating Cell Cycle Checkpoint

Cannabinoids are able to control various cell cycle checkpoints and prevent the progression of tumours [152]. Cyclin dependent kinases (CDKs) are protein kinases involved in regulating the cell cycle [153]. ER stress is known to impact cell cycle progression [154]. Treatment of breast cancer cells with THC puts stress on the ER and downregulates cyclin-dependent kinase 1 (CDK1) and cell division cycle (Cdc2), causing arrested G2-M transition [155]. CDK inhibitor p21 controls cell cycle progression, and its increased expression induces cell cycle arrest in both G1 and G2 phases [156]. ER stress inhibits cell cycle progression at the G1 phase, and cannabinoids, such as AEA, induce cell cycle arrest at the G1-S transition, via upregulation of CDK inhibitors p21 and p27, proteolysis of Cdc25 homologue A (Cdc25A) and inhibition of cyclin-dependent kinase 2 (Cdk2) [155]. AEA is also known to decrease the percentage of cells in G2-M phases. In melanoma, cell cycle is arrested at the G1-S transition, through the hypophosphorylation of the tumour suppressor retinoblastoma (Rb) and inhibition of AKT, and this occurs from the activation of receptors by cannabinoids [157]. Phosphorylation of Rb releases E2F transcription factor, which regulates cell cycle progression and is necessary for the gene transcription at G1-S [158]. This ability of cannabinoids to control cell cycle checkpoints could provide a therapeutic target for cancer treatment.

### 7.2. Role of Cannabinoids in Invasion, Metastasis and Angiogenesis

Along with the anti-proliferative and pro-apoptotic effects of cannabinoids on cancer cells, they also exhibit anti-invasive effects. Activation of receptors by cannabinoids blocks angiogenesis, tumour invasion and metastasis of cancer cells [159]. Stimulation of CB receptors by THC has shown anti-invasion activity in cervical and lung cancer models [160]. In prostate cancer cells, activation of CB1 by the endocannabinoid 2-AG inhibited invasion [160]. Angiogenesis is the recruitment of new blood vessels and is vital to tumour metastasis [161]. This largely comes down to tumour neovascularisation, which is important for tumour progression and can be suppressed by treatment with cannabinoids [161]. When inhibited, pro-angiogenic factors, such as vascular-endothelial growth factor (VEGF), placental growth factor and aniopoietin-2, are downregulated, preventing blood supply and nutrition to the tumour cells, thus inhibiting tumour growth and spread [161]. Decreased tumour vascularisation has been seen in glioma, breast and prostate cancer models [162,163,164].

### 7.3. Tumour-Immune Interactions

Cannabinoid receptors CB1 and CB2 are expressed on immune cells and may play a role in the regulation of the immune system. It is thought that cannabinoids could activate immune responses to averse the growth and dissemination of tumours. The underlying mechanism involves cannabinoid-induced upregulation of the intercellular adhesion molecule 1 (ICAM-1) on cancer cell surfaces and the following interaction with lymphocyte function-associated antigen 1 (LFA-1) on the outside of killer cells [165]. Recent evidence indicated that, in THC-treated animals, a decreased infiltration of skin tumour with macrophages and neutrophils is correlated with cancer reversion [166].

### 7.4. Cannabinoid-Induced Cell Death Mechanisms

**Figure 4:**
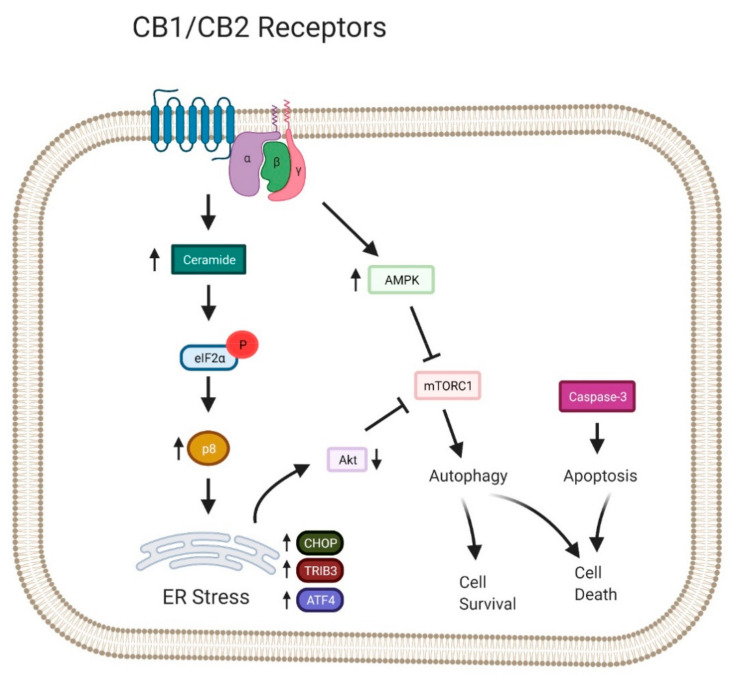
The Interplay between Autophagy and Apoptosis Induced by Cannabinoids 11. eIF2a, Eukaryotic Initiation Factor 2 alpha; p8, stress-regulated protein p8; CHOP, CCAAT/enhancer-binding protein homologous protein; TRIB3, pseudokinase tribbles homologue-3; ATF4, Activating transcription factor 4; ER, endoplasmic reticulum.

A multitude of in vitro and in vivo investigations have found cannabinoids to be effective in hindering tumour cell proliferation as well as inducing tumour cell death by initiating apoptosis and autophagy. Several studies have proposed a crosstalk linking apoptosis and autophagy, as these two pathways can either be two independent mechanisms or can work simultaneously [167]. There is an increasing interest in investigating the interaction and cooperation between apoptosis and autophagy as well as their potential therapeutic applications in different types of cancers (Figure 4).

According to a study by Munson et al., THC has shown inhibition of lung adenocarcinoma cell proliferation in vivo [168]. Although the antitumour potential of cannabinoids was demonstrated decades ago, the signalling pathways and mechanisms induced by cannabinoids in cancer, cell growth and cell death has remained inconclusive. The anticancer properties of cannabinoids largely depend on the interactions with different receptors and the activation of specific mechanisms in the different tumour systems [169]. Studies have indicated the same cell fate regardless the differing interactions of cannabinoids, their receptors, and the activation of dissimilar intracellular signalling cascades. For instance, the activation of CB1 or CB2 receptors results in diminished level of cyclic-AMP (cAMP) through the inhibition of adenylyl cyclase, consequently inducing canonical apoptosis [170]. Furthermore, several studies have established that the mitochondrial apoptotic pathway is initiated via the interaction of endocannabinoids AEA and TRPV1 in human neuroblastoma and lymphoma cells [171,172]. Induction of the apoptotic pathway obstructs cancer cell proliferation and is accompanied by accumulation of certain intracellular mediators including ceramide, reactive oxygen species (ROS) and some survival factors, which are also seen in the induction of autophagy. The accumulation of ceramide is facilitated via CB receptors and results in apoptosis in colon, glioma and pancreatic cancers [146,173,174,175]. It has been suggested that THC administration on glioma cells stimulates the de novo synthesis of ceramide and the biosynthesis of sphingolipids, consequently, leading to apoptotic cell death [146,176,177]. 

## 8. Cannabinoid-Induced Autophagy

Different studies have revealed the ability of cannabinoids to activate autophagy in different pathologies including cancer (Table 1). One of the most well-known examples of the interplay between apoptosis and autophagy is the ER stress response. Findings by Salazar et al. have proposed the activation of the ER stress-related signalling mechanism through the accumulation of ceramide in glioma cells [146]. Various other detrimental stimuli such as oxidative stress, misfolded proteins and infections also trigger an ER stress response as a defence mechanism [178]. The ER stress-related signalling pathway comprises of increased expression of stress-modulated protein p8 (p8), as well as the activation of downstream targets such as activating transcription factor 4 (ATF4), CCAAT/enhancer-binding protein homologous protein (CHOP) and TRIB3. The stimulation of the downstream targets leads to the activation of distinct cell death mechanisms. For instance, enhanced expression of CHOP results in mitochondria-dependent apoptosis; however, autophagy may also be activated by the substantial increase of CHOP levels that target the TRIB3 [179]. Autophagy can be induced by cannabinoids via the p8-regulated pathway. Studies have revealed that the upregulation of TRIB3 by THC inhibits the activity of AKT by decreasing its phosphorylation as well as its direct substrates, TSC2 and PRAS40. The inhibition of AKT results in the inhibition of the mammalian target of rapamycin complex 1 (mTORC1) and ultimately induces autophagic cell death [146,179]. Activation of the p8/TRIB3 pathway plays a prominent role in cannabinoid-induced autophagy in cancer cells. The reduction of tumour growth can be observed in dissimilar models of tumour xenografts, but not in p8-deficient tumour models that lack upregulation of the p8/TRIB3 pathway. Further evidence of the stimulation of p8/TRIB3 pathway was observed in glioma patients treated with THC [146,175]. A study by Salazar et al. has suggested that cannabinoid-induced autophagy endorsed apoptotic death of cancer cells [146]. Furthermore, the selective knockdown of autophagy-related genes such as ATG1 and ATG5 or Ambra1 inhibited THC-stimulated caspase-3 activation. Activation of caspase-3 plays a vital part in the apoptotic signalling pathway. This shows that autophagy-mediated cell death pathway precedes apoptosis in the cannabinoid antitumour response. Hence, the repression of autophagy inhibits cannabinoid-dependent apoptosis and cell death; conversely, the repression of apoptosis impairs only cannabinoid-dependent cell death, but not autophagy [13,146,147]. 

Mao et al. reported that cannabinoid receptor selective agonists enhanced the obstructed autophagic flux facilitated by the inflammatory mediators in the spinal cord, hence reducing bone cancer pain [189]. The impairment of autophagic flux in spinal neurons during bone cancer pain is indicated by the upregulation of LC3B-II/LC3B-I ratio as well as the accumulation of p62/SQSTM1 [9]. Alleviation of bone cancer pain is followed by the activation of autophagy flux and is indicated by decreased LC3B-II/LC3B-I ratio and decreased expression of p62/SQSTM1. Moreover, JWH-015 aids in the downregulation of the glia-derived inflammatory mediators like interleukin-1β (IL-1β) and interleukin-6 (IL-6) [189]. Another study has illustrated the contribution of cannabinoids in protecting liver from alcohol-stimulated steatosis [184]. Binge alcohol reduces autophagy in the liver, thus enhancing the development of liver steatosis. CYP2E1 mediated the alcohol-induced liver steatosis via mechanisms including the increased synthesis of ROS, the activation of MAPK JNK pathways as well as attenuated PPARα, which aid in the modulation of fatty acid oxidation [10,11]. CBD has been reported to protect the mice model from binge alcohol-induced steatosis through various pathways such as the repression of ROS production and the inactivation of MAPK JNK mechanism, thereby inducing autophagy [11]. 

Investigations have shown the efficacy of cannabinoid-induced autophagy in preventing cancer cell proliferation (Table 1). For instance, CBD treatment stimulated ER stress in breast cancer cells. As an ER stress response, the augmented phosphorylation of eukaryotic translation initiation factor 2, subunit 1 alpha (eIF2α) and reduced levels of phosphorylated mTORC1 have been observed [185]. THC and CBD (Sativex^®^) exhibited the induction of a non-canonical autophagic-facilitated apoptotic cell death in human melanoma cells, thus reducing melanoma viability, tumour growth and development [1]. Moreover, the combination of radiation therapy with a wide range of cannabinoids, like THC, CBD and synthetic cannabinoids such as nabilone, JWH-015 and CP55,940 have mediated the autophagy-induced tumour growth arrest and cell death [195]. Resorcinol derivative O-1663 has demonstrated similar results by inducing autophagy and apoptosis-mediated cell death in breast cancer cells, especially in metastatic cancer cells. The administration of a CB2 antagonist partially reversed the anti-metastatic activity in this case [190]. Another study reported the essential role of CB2 receptor activation in inducing the antitumorigenic effects of THC and JWH-015, via the TRIB3/AKT/mTORC1 mechanism in human HCC xenografts and cell lines [149]. In addition, the antitumour action of cannabinoids such as THC and JWH-015 has been demonstrated in HCC through induction of the autophagy flux and the activation of the PPARγ pathway. In vitro and in vivo studies have shown that both cannabinoids upregulate the intracellular level and activity of PPARγ, thereby inducing autophagy [196]. Moreover, recent work has revealed that treatment of human colon cancer cell lines and osteosarcoma cells with WIN55,212-2 stimulates ER stress-related signalling pathways and cell death. The WIN55,212-2 treatment recruits ER stress markers, including CHOP, TRIB3 and GRP78, and induces G2/M cell cycle arrest [191,192]. A study carried out by Dando et al. reported the significant increase of AMP/ATP levels with the use of synthetic cannabinoid inducing autophagy in pancreatic cancer cell lines [193]. 

Synthetic cannabinoids arachidonoyl cyclopropramide (APCA) and GW405833 have been revealed to suppress mitochondrial metabolism and initiate AMPK-dependent autophagy in pancreatic cancer cells [197]. Furthermore, the enhancement of ROS-mediated autophagy via the combination treatment of APCA and gemcitabine directly regulates the pancreatic cancer cell death [198,199]. Studies have investigated the antitumour effects of CBD alone and in synergism with a proteasome inhibitor termed bortezomib, to induce cell death in multiple myeloma (MM) cells [200]. The reduction of MM cell invasion by alleviating the chemokine receptors, known as CD147 and CXCR4, has been observed following administration of the CBD and THC combination treatment [201]. CBD and THC in combination are found to be synergistic with carfilzomib, a novel promising immune-proteasome inhibitor that aids in the suppression of MM cell migration and the elevation of MM cell death [201]. In addition, the upregulation of JNK1/2 and MAPK p38 signalling pathways is mediated by CBD, consequently resulting in the induction of autophagy in glioblastoma multiforme (GBM). Furthermore, CBD inhibited the AKT/mTOR pathway preventing the GBM proliferation [202]. There is an increased interest in the use of cannabinoids in combination with other anticancer drugs. The combination of cannabinoids with conventional chemotherapies and radiation therapies have largely reduced the drug cytotoxicity and increased therapeutic efficacy [203]. In addition, inhibition of GPR55 receptor by CBD decreased cancer cell proliferation and augmented gemcitabine efficacy in pancreatic cancer through MAPK signalling downregulation. The recent discovery of the potential dual targeting of autophagy and MEK in KRAS mutant cancer raised the intriguing possibility to use CBD in combination with autophagy inhibitors such as CQ or HCQ.

## 9. Conclusions and Future Perspectives

Autophagy is a natural process activated by various stress stimuli and is important in maintaining intracellular homeostasis. It is regulated by a variety of genes, hormones and growth factors. In cancer, autophagy has demonstrated a dual function, either aiding or inhibiting cancer cell proliferation and progression. In early stage tumours, autophagy has an antitumorigenic action, removing the tumour and damaged cellular constituents. In later stage tumours, however, autophagy can function as an alternative source of energy under detrimental cancer microenvironments, hence promoting cancer cell survival. The paradoxical properties of autophagy in cancer development have initiated investigations that focus on the autophagy regulatory mechanism, and how it can be used in anticancer therapies. 

Cannabinoids have also been investigated for their role in cancer, in the alleviation of chemotherapy side effects, and for their ability to reduce tumour growth and proliferation. In recent years, there has been an emerging interest in investigating synthetic cannabinoids to mediate autophagic cancer cell death, highlighting the potential application of cannabinoids as a novel targeted antitumour therapy. However, the consequences of cannabinoid-induced autophagy in cancer settings and the implications in tumour therapies are not completely understood; thus, further investigation into this mechanism is required. The combination of cannabinoids with conventional chemotherapies and radiation therapies have shown to largely reduce the drug cytotoxicity and increase therapeutic efficacy [203]. This is highly advantageous and could be very useful for improving the action of current cancer treatments.

Evidence has shown that the upregulation of autophagy may contribute to or increase drug resistance. Autophagy has been described as a protective mechanism that increases the survival rate of tumour cells. Several autophagy inhibitors, such as CQ and HCQ have been introduced to inhibit autophagy by altering the lysosomal pH, hence inhibiting accumulation of the autophagosome and the subsequent suppression of autophagic degradation. Therefore, the effects of cannabinoids on autophagy could be exploited in combined treatment with other existing chemotherapeutic agents to reduce resistance towards anticancer therapies. Initial phases of clinical trials are working to determine effective dosages that demonstrate an antitumorigenic action of cannabinoids. Studies are also investigating optimal administration methods. Future studies should focus on investigating the best combination of cannabinoids with particular attention towards drugs able to affect autophagy.

## Figures and Tables

**Table 1:** Cannabinoid-Induced Autophagy in Diseases.

Cannabinoid Classification	Cannabinoid	Diseases	Proposed Mechanism Mediating Autophagy	Reference
Phytocannabinoid	THC	Glioma	Induction of ER stress signalling pathways through ceramide accumulation Upregulation of p8 and activation of downstream targets CHOP, ATF4, and TRB3 Upregulation of TRB3 inhibiting AKT and mTOR, leading to autophagic cell death	Salazar et al., (2009) [140]
	THC + CBD	Melanoma	Regulated cell division	Bachari et al., (2020) [180]
	THC		Induction of G1-S cell cycle arrest through hypophosphorylation of Rb and inhibition of AKT	Blázquez et al., (2006) [181]
	THC + CBD	Multiple Myeloma	Downregulation of chemokine receptors such as CD147 and CXCR4	Nabissi et al., (2016) [182]
	THC + CBD + Carfilzomib (Immuno-proteasome inhibitor)		Irreversible adducts with β5i subunit of immuno-proteasome Increased cell death and inhibition of cell migration	
	THC + CBD + Bortezomib (Proteasome Inhibitor)		Alleviated cell proliferation and cell survival mechanism	Morelli et al., (2014) [183]
	CBD	Liver Steatosis	Decreased ROS production Downregulation of MAPK JNK pathway	Yang et al., (2014) [184]
	CBD	Breast Cancer	ER stress Increased eIF2α phosphorylation Decreased levels of mTORC1 G2 phase cell cycle arrest Cell growth arrest and cell death	Shrivastava et al., (2011) [185] Emery et al., (2014) [186]
	THC		G2 phase cell cycle arrest	Schoeman et al., (2020) [187]
	CBN			
	CBG			
	THC	Hepatocellular Carcinoma	Induction of PPARγ pathway	Vara et al., (2011) [131]
	CBD	Pancreatic Cancer	Increased levels of AMP/ATP Decreased mitochondrial metabolism	Dando et al., (2013) [169]
	CBD	Glioblastoma Multiforme	Induction of JNK1/2 and MAPK p38 signalling pathways Downregulation of AKT/mTOR mechanism	Ivanov et al., (2020) [188]
Synthetic Cannabinoid	JWH-015	Bone Cancer	Increased autophagic flux Decreased production of the glia-derived inflammatory mediators, IL-1β and IL-6	Mao et al., (2019) [189]
	JWH-015	Breast Cancer	Cell growth arrest and cell death	Emery et al., (2014) [186]
	CP55 940		Initiation of ROS production Inhibition of ID1 Reduced cell proliferation and invasion	Murase et al., (2014) [190]
	O-1663			
	JWH-015	Hepatocellular Carcinoma	Induction of the PPARγ pathway	Vara et al., (2011) [149]
	WIN55,212-2	Colon Cancer Osteosarcoma	ER stress Upregulation of CHOP, TRB3, GRP78 G2-M cell cycle arrest	Pellerito et al., (2014) [191] Notaro et al., (2014) [192]
	WIN55,212-2	Melanoma	Induction of G1-S cell cycle arrest through hypophosphorylation of Rb and inhibition of AKT	Blázquez et al., (2006) [181]
	arachidonoyl cyclopropamide	Pancreatic Cancer	Increased levels of AMP/ATP Decreased mitochondrial metabolism	Dando et al., (2013) [193]
	GW405833			
	WIN55,212-2 JWH133	Glioblastoma	Induction of autophagy and knockdown of autophagy genes Increased cannabinoid-induced apoptotic cell death	Ellert-Miklaszewska et al., (2021) [194]

ATF4, Activating transcription factor 4; CBD, cannabidiol; CBG, cannabigerol; CBN, cannabinol; CHOP, CCAAT/enhancer-binding protein homologous protein; ER, endoplasmic reticulum; eIF2a, eukaryotic translation initiation factor 2, subunit 1 alpha; JNK, jun *N*-terminal kinase; mTOR, mammalian target of rapamycin; MAPK, mitogen-activated protein kinase; PPARγ, peroxisome proliferator-activated receptor gamma; AKT, protein kinase B; Rb, retinoblastoma; TRIB3, pseudokinase tribbles homologue-3; p8, stress-modulated protein p8; THC, Δ^9^-tetrahydrocannabinol; IL-1β, interleukin 1 beta; IL-6, interleukin 6; GRP78, glucose-regulated protein 78; ROS, reactive oxygen species; ID1, Inhibitor of DNA Binding 1; CXCR4, C-X-C chemokine receptor type 4; CD147, basigin; JWH015, JWH133, CP55 940, O-1663 and GW405833 are synthetic cannabinoid receptor 2 agonists; WIN55,212-2, is a synthetic cannabinoid receptor 1 and PPAR agonist.

## Data Availability

Databases MEDLINE/PubMed, Google Scholar and EMBASE were searched for studies on the autophagy pathway, cannabinoids, and their effect on cancer. The search terms ‘autophagy’, ‘cannabinoids’, ‘cancer’, ‘regulation’ and ‘therapy’ were used. Publications from the reference lists of recovered articles were also reviewed. Articles were published between 1967 and 2021. Articles not using English language and non-peer reviewed were excluded. The final database search was performed in February 2021.

## Abbreviations

Activating transcription factor 4	ATF4
adenosine monophosphate-activated protein kinase	AMPK
arachidonyl-2′-chloroethylamide	ACEA
arachidonoyl cyclopropramide	APCA
2-arachidonoylglycerol	2-AG
autophagy-related	Atg
bax interacting factor-1	Bif-1
B-cell lymphoma 2	Bcl2
Bcl-2 homology 3 domain	BH3
BECN1-regulated autophagy protein 1	Ambra1
Camptothecin	CPT
Cannabidiol	CBD
cannabinoid receptor 1	CB1
cannabinoid receptor 2	CB2
cdc25 homologue A	Cdc25A
CCAAT/enhancer-binding protein homologous protein	CHOP
cell division cycle	Cdc2
chloroquine	CQ
central nervous system	CNS
extracellular matrix, cyclin dependent kinases	CDKs
cyclin-dependent kinase 1	CDK1
cyclin-dependent kinase 2	Cdk2
cyclic-AMP	cAMP
ECM; eukaryotic translation initiation factor 2, subunit 1 alpha	eIF2α
Endoplasmic reticulum	ER
extracellular-regulated kinase	ERK
5–Fluorouracil, fatty acid amide hydrolase	FAAH; 5FU
genetically engineered mouse models	GEMMs
glioblastoma multiforme	GBM
G-protein coupled receptor	GPCR
G-protein coupled receptor 55	GPR55
Hydroxychloroquine	HCQ
hypoxia-induce factor-1 alpha	HIF-1α
intercellular adhesion molecule 1	ICAM-1
jun *N*-terminal kinase	JNK
lymphocyte function-associated antigen 1	LFA-1
mammalian target of rapamycin	mTOR
mammalian target of rapamycin complex 1	mTORC1
3-methyladenine	3-MA
microtubule-associated protein light chain 3	LC3B
mitogen-activated protein kinases	MAPKs
monoacylglycerol lipase	MAGL
multiple myeloma	MM
peroxisome proliferator-activated receptors	PPARs
phosphoinositide 3-kinase	P13K
*N*-arachidonoylethanolamine	AEA
non-small-cell lung cancer	NSCLC
*N*-oleoylethanolamine	OEA
*N*-palmitoylethanolamine	PEA
protein kinase B	AKT
pseudokinase tribbles homologue-3	TRIB3
reactive oxygen species	ROS
retinoblastoma	Rb
sequestosome-1	SQSTM1
soluble *N*-ethylmaleimide-sensitive factor attachment protein receptor	SNARE
stress-modulated protein p8	p8
Δ^9^-tetrahydrocannabinol	THC
transient receptor potential vanilloid type 1	TRPV1
transient receptor potential vanilloid type 2	TRPV2
tuberous sclerosis protein 2	TSC2
UV radiation resistance-associated gene	UVRAG
vascular-endothelial growth factor	VEGF
